# Differential Effects of Accumbens Core vs. Shell Lesions in a Rat Concurrent Conditioned Place Preference Paradigm for Cocaine vs. Social Interaction

**DOI:** 10.1371/journal.pone.0026761

**Published:** 2011-10-26

**Authors:** Michael Fritz, Rana El Rawas, Sabine Klement, Kai Kummer, Michael J. Mayr, Vincent Eggart, Ahmad Salti, Michael T. Bardo, Alois Saria, Gerald Zernig

**Affiliations:** 1 Experimental Psychiatry Unit, Department of General Psychiatry and Social Psychiatry, Center for Psychiatry and Psychotherapy, Medical University Innsbruck, Innsbruck, Austria; 2 Institute for Neuroscience, Medical University Innsbruck, Innsbruck, Austria; 3 Center for Drug Abuse Research Translation, University of Kentucky, Lexington, Kentucky, United States of America; University of Granada, Spain

## Abstract

**Background:**

A main challenge in the therapy of drug dependent individuals is to help them reactivate interest in non-drug-associated activities. Among these activities, social interaction is doubly important because treatment adherence itself depends on it. We previously developed a rat experimental model based on the conditioned place preference (CPP) paradigm in which only four 15-min episodes of social interaction with a gender- and weight-matched male conspecific (i) reversed CPP from cocaine to social interaction despite continuing cocaine training and (ii) prevented the reinstatement of cocaine CPP. In the present study, we investigated if the two subregions of the nucleus accumbens (Acb), i.e., the core (AcbC) and the shell (AcbSh), would differentially affect CPP for cocaine vs social interaction.

**Methodology/Principal Findings:**

Animals were concurrently trained for CPP pairing cocaine with one compartment and social interaction with the other (i.e., mutually exclusive stimulus presentation during training). Excitotoxic lesioning of the AcbC or the BLA shifted CPP toward social interaction, whereas AcbSh inactivation shifted CPP toward cocaine.

**Conclusions:**

Overall, our findings suggest that inactivation of the AcbC or the BLA is sufficient to shift CPP away from a drug of abuse toward social interaction. Lesioning the AcbSh produced the opposite effect.

## Introduction

Rekindling the interest of a recovering drug dependent individual toward non-drug-associated activities remains one of the biggest challenges in the therapy of substance-use disorders [1], [2]. Among these “alternative”, i.e., non-drug-associated, activities, social interaction is of special importance because psychotherapy, one of the mainstays of therapy, depends on the addict's ability to engage in beneficial social interactions (again) and because dyadic social interaction is arguably the biggest single beneficial factor of psychotherapy itself [3], [4]. To study the neurobiological basis of the reallocation of behavior away from the drug of abuse toward social interaction, we developed a rat experimental model based on the conditioned place preference (CPP) paradigm in which only four 15-min episodes of social interaction with a gender- and weight-matched male conspecific (i) reversed CPP from cocaine to social interaction despite continuing cocaine training and (ii) prevented the reinstatement of cocaine CPP [5]. The reversal of CPP from cocaine to social interaction was enhanced by the sigma1 receptor antagonist BD1047 with an intraperitoneal (i.p.) ED50 of 0.0036 mg/kg [6]. The CPP reversal by social interaction was paralleled by a reversal of the cocaine conditioning-induced Zif268 activation in the medial nucleus accumbens shell (AcbSh) and, to a lesser degree, in the anterior commissure-surrounding core (AcbC; not significant), the central (CeA) and basolateral (BLA) amygdala, and the ventral tegmental area (VTA) [5].

Thus, in accordance with a wealth of data (see, e.g., [7], [8]), our previous findings [5] had suggested that the core and shell subregions of the nucleus accumbens may differentially affect CPP for cocaine vs CPP for social interaction. Therefore, the present study was designed to further investigate the differential roles of the core and shell subregions of the Acb and of the BLA as a major input into the Acb [9], [10] by selective excitotoxic lesioning with quinolinic or ibotenic acid [11] and subsequent concurrent acquisition and expression of cocaine- vs social interaction CPP. The present findings indicate that the balance of the incentive salience of the social interaction- vs cocaine-associated contextual stimuli (CSs) of the CPP procedure can be shifted toward social interaction by lesioning the AcbC or the BLA, whereas lesioning the AcbSh shifted the balance toward cocaine CPP, i.e., that neuron ensembles in the two accumbal subregions differentially encode the incentive salience of the non-drug stimulus social interaction vs the drug stimulus cocaine.

## Results

### Effect of lesions of the AcbC, AcbSh, or BLA on the concurrent acquisition and expression of cocaine CPP

Our testable hypothesis posited that the two subregions of the Acb, i.e., the core (AcbC) and the shell (AcbSh), would differentially affect the incentive salience of a drug reward vs that of a natural reinforcer, i.e., social interaction. The most parsimonious approach to test this hypothesis was to let rats acquire conditioned place preference (CPP) for cocaine as the prototypical drug of abuse and for social interaction as the alternative reward in a concurrent manner, to selectively inactivate either the AcbC or AcbSh and to investigate if this selective lesioning would shift the balance of CPP from the drug reward to the social interaction reward or vice versa. Lesioning the BLA, a structure with strong projections to the AcbC, was hypothesized to produce essentially the same changes as lesioning the AcbC.

**Figure 1:**
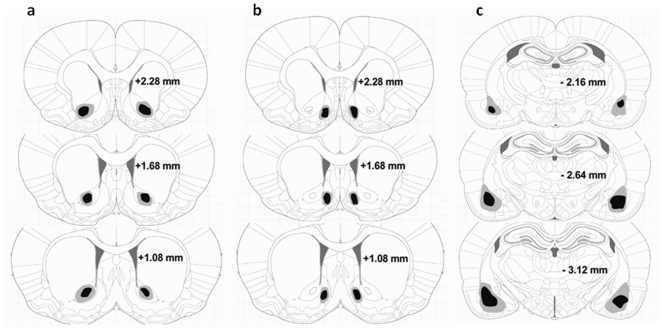
Location of the excitotoxic lesions of the nucleus accumbens core and shell and the basolateral amygdala. Shown is the extent of the (a) quinolinic lesions of the AcbC, (b) ibotenic acid lesions of the AcbSh, and (c) quinolinic lesions of the BLA. The black and grey areas indicate the minimum and maximum spread of the excitotoxins as assessed by inspection of the slices. Coronal sections are +2.28 mm anterior through +1.08 mm posterior to Bregma (A and B) and −2.16 mm anterior through −3.12 mm posterior to Bregma (C) according to the atlas of Paxinos and Watson [17].

**Figure 2:**
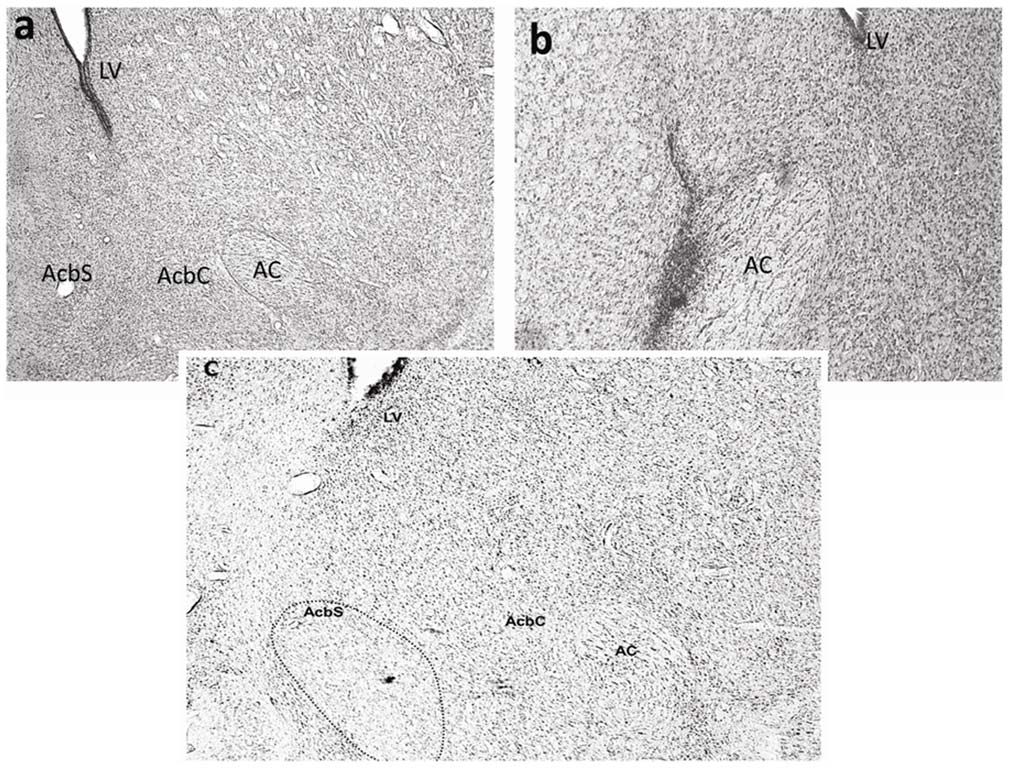
Representative photomicrographs showing cresyl violet-stained coronal sections through the nucleus accumbens of excitotoxin-lesioned and vehicle-treated rats. (a) Cresyl violet staining through the Acb of a vehicle-treated rat showing the same density of staining in the shell and the core of the Acb. (b) Cresyl violet staining of an AcbC lesion. Neurons in the AcbC have been replaced by densely stained neurons indicating gliosis around the surface of the anterior commissure. The staining in the AcbSh is preserved. (c) Cresyl violet staining of a partial AcbSh lesion, showing the loss of staining in the shell region and the preservation of the staining in the AcbC. Abbreviations: AC, anterior commissure; AcbSh, nucleus accumbens shell; AcbC, nucleus accumbens core; LV, lateral ventricle.

**Figure 3:**
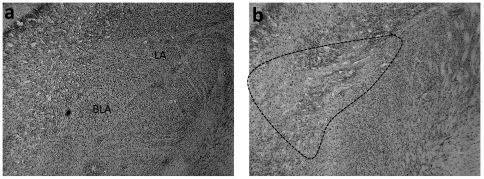
Representive photomicrographs showing cresyl violet-stained coronal sections through the basolateral amygdala of lesioned and sham control rats. (a) Cresyl violet staining of the BLA of a vehicle-treated rat and (b) a lesioned animal. The lesioned area is indicated by dotted lines. Lesions caused significant neuronal loss to the basal and lateral amygdaloid nucleus. The staining in the central and the medial nucleus of the amygdala was preserved. Abbreviations: LA, lateral amygdala; BLA, basolateral amygdala.

Figures 1, 2, 3 show the location and extent of the bilateral excitotoxic lesions of the three areas of interest (AcbC, AcbSh, and BLA) as compared to tissue from animals which were surgically implanted with cannulae but only received vehicle (“sham”).

**Figure 4:**
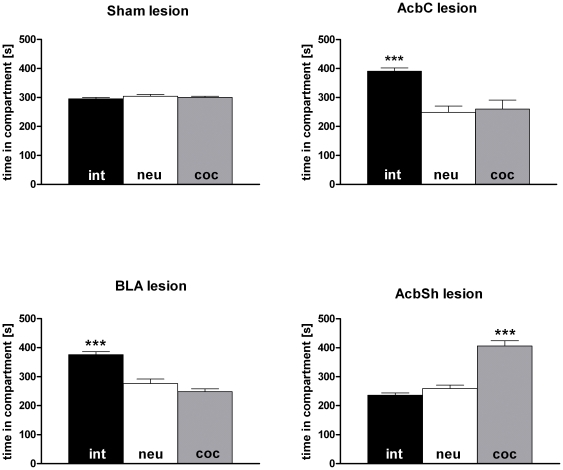
Lesions to nucleus accumbens core vs shell shifts preference for social interaction vs cocaine. Shown are times spent in the stimulus-associated compartment during the CPP test (total 900 s) by rats that had undergone concurrent CPP training for social interaction (int) and cocaine (coc). Time spent in the neutral compartment of the 3-chamber CPP apparatus is designated “neu”. All times are presented as mean ± SEM. Before the concurrent CPP training, rats either received sham stereotaxic surgery including intracerebral injection of vehicle (sham lesion, n* = *12) or received bilateral excitotoxic lesions of the AcbC (n* = *6), the AcbSh (n* = *6), or the BLA (n* = *7). P values (ANOVA) for times spent in the respective chambers were P* = *0.46 for the sham-treated rats, P* = *0.0007 for the AcbC-lesion rats, P*<*0.0001 for the AcbSh-lesioned rats, and P*<*0.0001 for the BLA-lesioned animals. The two experimenters (MF and SK) were blind to the treatment.

In order to replicate our previous findings on the concurrent acquisition of social interaction- vs cocaine CPP [5] and to control for possible surgery effects, rats received bilateral intracerebral injections of vehicle in either the AcbC, AcbSh, and BLA. Subsequent CPP training of the vehicle-treated rats yielded times spent in the social interaction (int)- or cocaine (coc)-associated chamber or middle (neutral, neu) chamber that did not differ among vehicle-injected brain region (AcbC, n* = *3; AcbSh, n* = *6; and BLA, n* = *3; one-factor ANOVA, P* = *0.52 for int, P* = *0.50 for neu, and P* = *0.88 for coc). Thus, all three vehicle groups were pooled for subsequent analysis (Fig. 4, “sham lesion”, total n = 12). During the CPP test, the vehicle-treated animals spent equal times in the previously social interaction-associated compartment, previously cocaine-associated compartment, and the neutral compartment (ANOVA, P* = *0.46). Thus, the present sham-lesioning results replicate previous findings by our group (fig. 2a of [5]) indicating that since both stimuli (15 min dyadic social interaction vs 15 mg/kg i.p. cocaine) can produce equally strong CPP, there is no net CPP for either of them. If, however, the AcbC was excitotoxically lesioned before CPP training, net overall CPP for the social interaction-associated compartment developed (Fig. 4; n* = *6; ANOVA, P* = *0.0007; Tukey's post-hoc test, P<0.01 for int vs coc). Similarly, lesioning the BLA led to a CPP for the social interaction-associated compartment (Fig. 4; n* = *7; ANOVA, P*<*0.0001; Tukey's post-hoc test, P<0.001 for int vs coc). In contrast, lesioning the AcbSh led to a CPP for the cocaine associated chamber (Fig. 4; n* = *6; ANOVA, P*<*0.0001; Tukey's post-hoc test, P<0.001 for int vs coc).

## Discussion

If rats were concurrently conditioned for place preference by pairing cocaine with one compartment and social interaction with the other (i.e., mutually exclusive stimulus presentation during training), pre-acquisition lesioning the accumbens core (AcbC) or the basolateral amygdala (BLA) shifted the animals' preference toward social interaction, whereas a bilateral shell (AcbSh) lesion shifted the preference toward cocaine CPP. Our findings thus suggest that the incentive salience of drug-associated conditioned contextual stimuli is – at least preferentially – mediated by the core and the BLA, whereas the incentive salience of non-drug-associated conditioned contextual stimuli seems to be more localized in the shell subregion of the Acb.

These conclusions, based on a ***concurrent*** CPP approach - which only assesses the ***balance*** of the incentive salience of social interaction- vs cocaine-associated contextual stimuli in the CPP paradigm, extend previous findings by other groups who have used CPP to assess the incentive salience of drug-associated contextual stimuli. Neisewander and colleagues [12] blocked acquisition of cocaine CPP by bilateral BLA lesions with N-methyl-D-aspartic acid (NMDA). Similarly, Sellings and Clarke [13] inhibited acquisition of amphetamine CPP by bilateral 6-hydroxydopamine lesion of the AcbC but not AcbSh [13]. In this latter report, the number of remaining dopamine transporters (DATs) in the AcbSh correlated with the extent of amphetamine CPP, whereas the number of remaining DATs in the AcbC correlated with the extent of remaining amphetamine-induced locomotor stimulation.

Once acquired, CPP to drugs of abuse seems to be differently affected by lesioning. Specifically, if Neisewander and colleagues lesioned the BLA with NMDA after cocaine CPP had been acquired, extinction of cocaine CPP was slowed down [12]. Wang and coworkers also found that morphine-induced reinstatement of extinguished morphine CPP was inhibited by lesions of the AcbSh but not AcbC. Based on the explanation given by Neisewander and colleagues [12], it is possible that post-acquisition lesions of the BLA or the AcbSh disrupted the extinction of drug CPP by impairing the assessment of the current incentive salience [2] of previously drug-paired stimuli, whereas the AcbC, which is downstream of the AcbSh [8], may be more involved in the locomotor execution [13] of CPP behavior. Our findings (Figure 4) suggest further that, with respect to the Acb subregions, cocaine CPP is predominantly represented in such an AcbC locomotor execution area and that social interaction, predominantly involving the AcbSh, may counteract this “automatic” motoric drug-driven behavior, a notion consistent with the “go-circuit vs stop-circuit” neural networks discovered by Peters, Kalivas and colleagues [14].

Lesioning the BLA (Figure 4) produced the same CPP preference shift away from cocaine toward social interaction as the AcbC lesions, suggesting that, overall, input of the BLA into the AcbC may be more important than the BLA's input into the AcbSh for the mediating cocaine CPP. As different neuronal ensembles of the basal amygdaloid complex project to the AcbC and/or AcbSh in intricate patterns [9], [10], [15], the Acb itself being a highly complex and heterogeneous structure [8], future studies will have to be performed at the single neuron level to identify which neuron ensembles encode the respective incentive salience of the two vastly different stimuli (i.e., cocaine vs social interaction).

In conclusion, our findings demonstrate that inactivation of the core of the nucleus accumbens or the basolateral amygdala can shift the balance of conditioned preference for contextual stimuli away from the drug of abuse cocaine toward social interaction, an effect that is highly desirable for the therapy of substance dependent individuals.

## Materials and Methods

### Animals

Male Sprague Dawley rats (n* = *39) were obtained from the Research Institute of Laboratory Animal Breeding of the Medical University Vienna (Himberg, Austria) and had to weigh 150 −250 g (corresponding to an age of 6 – 8 weeks, which can be considered early adulthood [16]) to be advanced to the CPP experiments. All animals were housed in groups of six rats until the surgical intervention, from which time on they were kept in single cages at a constant room temperature of 24°C and had *ad libitum* access to tap water and pelleted chow (Tagger, Austria). The rats were kept at a 12-h light/dark cycle with the lights on from 0800 h to 2000 h. Experiments were conducted during the light period of the cycle. The animals used in this study were cared for in accordance with the guidelines of the National Institutes of Health Animal Care and Use Program and the NIDA-IRP Animal Program, and the present experiments were approved by the Austrian National Animal Experiment Ethics Committee.

### Surgery

Rats were deeply anesthetised with isoflurane and secured in a Stoelting stereotaxic instrument (www.stoelting.com). All stereotaxic coordinates were taken from the atlas of Paxinos and Watson [17]. Following the excitotoxic lesioning protocol by Everitt, Robbins and coworkers (see table 1 of [11]), 10 animals received bilateral nucleus accumbens core (AcbC) lesions induced by an injection of 0.5 microliter of quinolinic acid (0.09 mol/l) over 3 minutes at the following coordinates: anteroposterior (AP) +1.2 mm, mesolateral (ML) ±1.8 mm, and dorsoventral (DV) −7.1 mm. The cannula was removed 2 min after the end of the injection to ensure appropriate diffusion. Seven rats received bilateral nucleus accumbens shell (AcbSh) lesions by infusing ibotenic acid (0.06 mol/l) for an overall of four min at three different sites. The coordinates were as follows: (1) AP +1.6 mm, ML±1.1 mm, DV −7.9 mm; (2) AP +1.6 mm, ML±1.1 mm, DV −6.9 mm; (3) AP +1.6 mm, ML±1.1 mm, DV −6.4 mm. The volume, injection and diffusion time for the three sides were (1) 0.1 microliter, 1 min, 1 min; (2) 0.1 microliter, 1 min, 1 min; (3) 0.2 microliter, 2 min, 1 min. The basolateral amygdala (BLA) lesion group (*n = *9) received bilateral injections of 0.09 mol/l quinolinic acid at two locations, again following the protocol by Everitt and coworkers (see table 1 of [18]). The injections were placed at (1) AP −2.3 mm, ML±4.6 mm, DV −7.3 mm; (2) AP −3 mm; ML±4.6 mm, DV −7.3 mm. At both positions 0.3 microliter were infused over 2 min. The needle remained at place for additional two minutes to ensure diffusion. All neurotoxin infusions were given through a single burr hole using a microsyringe (www.hamiltoncompany.com). Quinolinic- or ibotenic acid were dissolved and buffered to pH 7.3 – 7.4 in 0.1 mol/l phosphate buffered saline (PBS; vehicle). Sham-treated animals received only bilateral vehicle injections. Only 1 of 39 rats had to be euthanized due to observable intense postsurgical pain reactions. After the surgery, all rats were placed in single cages and left for 7 – 10 days to recover before proceeding to the behavioral experiments. The concurrent CPP acquisition experiments were performed by two independent experimenters who were blind to the treatment.

### Conditioned place preference apparatus

The CPP apparatus consisted of three compartments. The middle (neutral) compartment (10×30×30 cm) had grey walls and a grey floor. Two doorways led to the two conditioning compartments (25×30×30 cm each) with walls showing either vertical or horizontal black-and-white stripes of the same overall brightness and with stainless steel floors containing either holes (diameter 0.5 cm) or 15 slits (5×0.5 cm each). Time spent in each compartment was taken with hand timers in the lesioning experiments. In the subsequent cocaine PT experiments, behavior in the CPP apparatus during the cocaine CPP test was recorded with a video camera and analysed offline. There were no grossly observable differences between video-analyzed (present study) and hand-timed [19] cocaine CPP data; not shown). If the added-up times for all three compartments were less than the total 900 s of the test session, the missing time was distributed equally among the three chambers to avoid any bias. After every single rat, the apparatus was cleaned with a 70% camphorated ethanol solution.

### Place conditioning procedure

For the concurrent acquisition of CPP to social interaction vs cocaine, the conditioning procedure comprised a pretest session on day 1, eight consecutive training days (alternate-day-design, one 15-min training session per day, a total of 4 training sessions each for social interaction or cocaine, social interaction exposure on day 1, cocaine exposure on day 2, social interaction on day 3, cocaine on day 4 etc.), and a CPP test on day 10. Training- and CPP test session length were of equal duration, i.e., 15 min* = *900 s. Preference for any of the two conditioning chambers was assessed in a pretest session and was declared if the rat spent more time in one of the conditioning chambers. The initially nonpreferred chamber was subsequently paired with social interaction or cocaine in a within-group counterbalanced design (rat 1, social interaction; rat 2, cocaine etc.). Social interaction consisted of a 15-min episode in one of the CPP compartments with a weight- and gender-matched conspecific within the confines of one conditioning chamber of the CPP box. The conspecific remained the same dyadic partner for a total of 4 episodes and was also singly housed during the whole duration of the experiment. Gross observation indicated that only “agonistic” (i.e., “friendly”) social interaction, i.e., touching, crawling under, and grooming occurred, whereas “antagonistic” (i.e., threatening, boxing, fighting, biting etc.) behavior was not observed during the training sessions. Immediately before the start of each social interaction CPP training session, the rat was administered an intraperitoneal (i.p.) injection of 1 ml/kg saline to control for possible handling- and i.p. injection effects. Cocaine (15 mg/kg pure base, also given i.p. immediately before the start of the session) and social interaction were always offered in a mutually exclusive compartment. CPP testing was conducted at least 24 h after the last cocaine exposure, insuring that cocaine was eliminated from the rat's brain [20] at the beginning of the CPP test.

### Cresyl violet staining

After the CPP test, rats that had received bilateral stereotaxic cannulae were deeply anesthetized using isoflurane and intracardially perfused with 0.1 mol/l phosphate buffered saline (PBS; pH 7.4) followed by 4% paraformaldehyde (PFA) dissolved in 0.1 mol/l PBS. Brains were removed and postfixed in 4% PFA overnight, stored in 30% sucrose at 4°C until the brain sank (indicating sufficient penetration of sucrose into the brain tissue), and subsequently at −80°C until sectioning. Coronal sections (40 µm) were cut using a Leica CM3050 S cryostat (www.leica.com), throughout the full extent of the lesioned area. Every third section was taken, mounted on a gelatin coated glass slide, and stained with cresyl violet (0.1 mol/l sodium acetate, 2% acetic acid, 0.02 mol/l cresyl violet acetate in distilled water, pH 3.7; www.sigmaaldrich.com). Lesions were verified using a Zeiss optical microscope equipped with a camera (Axioplan 2 Imaging) interfaced to a PC. Rats (7 of 38 total) that did not show a lesion in the targeted areas were not included in the behavioral analysis.

### Statistical Analysis

All results are presented as group means ± SEM. Behavioral results were analyzed using one-factor (time spent in the respective CPP chamber) analyses of variance (ANOVA) followed by the Tukey's post-hoc test. Differences were considered significant at p<0.05. All statistical tests were performed with GraphPad Prism® 4.

### Drugs

Cocaine HCl was generously provided to G.Z. by the National Institute on Drug Abuse (NIDA). Cocaine was administered as 15 mg/kg pure base in a volume of 1 ml/kg saline. All other research compounds were obtained commercially.

## References

[1] Zernig G, Saria A, Kurz M, O'Malley SS, Hollinger MA (2000). Handbook of alcoholism;.

[2] Zernig G, Ahmed SH, Cardinal RN, Morgan D, Acquas E (2007). Explaining the escalation of drug use in substance dependence: Models and appropriate animal laboratory tests.. Pharmacology.

[3] Grawe K (1997). Research-informed psychotherapy.. Psychotherapy Res.

[4] Berns U (2004). Spezifische psychoanalytische Interventionen. Kaum wirksam, doch unverzichtbar?. Forum der Psychoanalyse 20.

[5] Fritz M, El Rawas R, Salti A, Klement S, Bardo MT (2011). Reversal of cocaine-conditioned place preference and mesocorticolimbic Zif268 expression by social interaction in rats.. Addict Biol.

[6] Fritz M, Klement S, El Rawas R, Saria A, Zernig G (2011). Sigma1 receptor antagonist BD1047 enhances reversal of conditioned place preference from cocaine to social interaction.. Pharmacology.

[7] Pontieri FE, Tanda G, DiChiara G (1995). Intravenous cocaine, morphine, and amphetamine preferentially increase extracellular dopamine in the “shell” as compared with the “core” of the rat nucleus accumbens.. ProcNatlAcadSci.

[8] Zahm DS (2000). An integrative neuroanatomical perspective on some subcortical substrates of adaptive responding with emphasis on the nucleus accumbens.. Neurosci BiobehavRev.

[9] Wright CI, Beijer AV, Groenewegen HJ (1996). Basal amygdaloid complex afferents to the rat nucleus accumbens are compartmentally organized.. JNeurosci.

[10] Aggleton JP (2003). The amygdala.. A functional analysis.

[11] Murphy ER, Robinson ES, Theobald DE, Dalley JW, Robbins TW (2008). Contrasting effects of selective lesions of nucleus accumbens core or shell on inhibitory control and amphetamine-induced impulsive behaviour.. EurJ Neurosci.

[12] Fuchs RA, Weber SM, Rice HJ, Neisewander JL (2002). Effects of excitotoxic lesions of the basolateral amygdala on cocaine-seeking behavior and cocaine conditioned place preference in rats.. Brain research.

[13] Sellings LH, Clarke PB (2003). Segregation of amphetamine reward and locomotor stimulation between nucleus accumbens medial shell and core.. The Journal of neuroscience: the official journal of the Society for Neuroscience.

[14] Peters J, LaLumiere RT, Kalivas PW (2008). Activity in infralimbic cortex suppresses cocaine relapse.. J Neurosci.

[15] Mashhoon Y, Tsikitas LA, Kantak KM (2009). Dissociable effects of cocaine-seeking behavior following D1 receptor activation and blockade within the caudal and rostral basolateral amygdala in rats.. Eur J Neurosci.

[16] Spear LP (2000). The adolescent brain and age-related behavioral manifestations.. NeurosciBiobehavRev.

[17] Paxinos G, Watson C (2007). The rat brain in stereotaxic coordinates..

[18] Parkinson JA, Robbins TW, Everitt BJ (2000). Dissociable roles of the central and basolateral amygdala in appetitive emotional learning.. EurJNeurosci.

[19] Zernig G, Fritz M, Klement S, El Rawas R, Saria A (2011). Social interaction- vs cocaine conditioned place preference is associated with a differential activation of nucleus accumbens core cholinergic interneurons.. Drug Alcohol Dep.

[20] Crespo JA, Sturm K, Saria A, Zernig G (2006). Activation of muscarinic and nicotinic acetylcholine receptors in the nucleus accumbens core is necessary for the acquistion of drug reinforcement.. J Neurosci.

